# Rational design of *Striga hermonthica*-specific seed germination inhibitors

**DOI:** 10.1093/plphys/kiab547

**Published:** 2021-11-27

**Authors:** Randa A Zarban, Umar F Shahul Hameed, Muhammad Jamil, Tsuyoshi Ota, Jian You Wang, Stefan T Arold, Tadao Asami, Salim Al-Babili

**Affiliations:** 1 Division of Biological and Environmental Sciences and Engineering, King Abdullah University of Science and Technology, The BioActives Lab, Thuwal, 23955-6900, Saudi Arabia; 2 Division of Biological and Environmental Sciences and Engineering, King Abdullah University of Science and Technology, Computational Bioscience Research Center, Thuwal, 23955-6900, Saudi Arabia; 3 Graduate School of Agricultural and Life Sciences, The University of Tokyo, Tokyo, Japan; 4 Centre de Biochimie Structurale, CNRS, INSERM, Université de Montpellier, Montpellier, 34090 France

## Abstract

The obligate hemiparasitic weed *Striga hermonthica* grows on cereal roots and presents a severe threat to global food security by causing enormous yield losses, particularly in sub-Saharan Africa. The rapidly increasing Striga seed bank in infested soils provides a major obstacle in controlling this weed. *Striga* seeds require host-derived strigolactones (SLs) for germination, and corresponding antagonists could be used as germination inhibitors. Recently, we demonstrated that the common detergent Triton X-100 is a specific inhibitor of *Striga* seed germination by binding noncovalently to its receptor, *S. hermonthica* HYPO-SENSITIVE TO LIGHT 7 (ShHTL7), without blocking the rice (*Oryza sativa*) SL receptor DWARF14 (OsD14). Moreover, triazole ureas, the potent covalently binding antagonists of rice SL perception with much higher activity toward OsD14, showed inhibition of *Striga* but were less specific. Considering that Triton X-100 is not suitable for field application and by combining structural elements of Triton and triazole urea, we developed two hybrid compounds, KK023-N1 and KK023-N2, as potential *Striga*-specific germination inhibitors. Both compounds blocked the hydrolysis activity of ShHTL7 but did not affect that of OsD14. Binding of KK023-N1 diminished ShHTL7 interaction with *S. hermonthica* MORE AXILLARY BRANCHING 2, a major component in SL signal transduction, and increased ShHTL7 thermal specificity. Docking studies indicate that KK023-N1 binding is not covalent but is caused by hydrophobic interactions. Finally, in vitro and greenhouse tests revealed specific inhibition of *Striga* seed germination, which led to a 38% reduction in *Striga* infestation in pot experiments. These findings reveal that KK023-N1 is a potential candidate for combating *Striga* and a promising basis for rational design and development of further *Striga*-specific herbicides.

## Teaser

Designed strigolactone antagonists inhibit *Striga* seed germination.

## Introduction

*Striga hermonthica*, commonly known as “purple witchweed,” is a root-parasitic plant and considered one of the major biotic constraints to food production in sub-Saharan Africa (Musselman et al., 2001; Parker, 2009; Pennisi, 2010; Rodenburg et al., 2016). The infestation of cereals, such as maize (*Zea mays*), pearl millet, sorghum, and upland rice by *Striga* results in enormous yield losses. It can lead to complete crop failure, imposing a significant burden on the food security of more than 300 million people and causing economic costs of around seven billion US dollars annually (Gressel et al., 2004; Ejeta, 2007; Atera et al., 2012). It is estimated that 50 million hectares of arable lands in Africa are infested by *Striga* (Gressel et al., 2004; Ejeta, 2007). *Striga* is characterized by exceptional fertility that gives rise to thousands of seeds per plant with 10–15 years of longevity in soil (Ejeta, 2007; Jamil et al., 2012), building up large seed reservoirs in infested fields and appearing as one of the main obstacles in the long-term management of *Striga* (Kountche et al., 2019). Albeit the employment of a number of mechanical, cultural, biological, and chemical control measures by local farmers, *Striga* is still a major problem for agriculture in sub-Saharan Africa (Eplee and Norris, 1995; Joel et al., 2007; Aly, 2012; Jamil et al., 2021). Hence, there is an urgent demand for control strategies that can deplete accumulated seed bank in infested soils or specifically inhibit the germination of *Striga* seeds (Ejeta, 2007; Cardoso et al., 2011; Zwanenburg et al., 2016; Jamil et al., 2018, 2019, 2020; Kountche et al., 2019).

Like other root parasitic plants, *Striga* is an obligate parasite that needs a host to survive (Xie et al., 2010). To ensure that emerging seedlings have access to a suitable host, seeds of these weeds germinate only upon sensing host-released signaling molecules, mainly strigolactones (SLs; Bouwmeester et al., 2003; Al-Babili and Bouwmeester, 2015). SLs are a group of carotenoid derived plant hormones consisting of a lactone ring (D-ring) coupled with an enol ether bridge to a second variable moiety (Jia et al., 2018; Moreno et al., 2021; Wang et al., 2021). They are released into the rhizosphere to attract symbiotic mycorrhizal fungi and play pivotal roles in various aspects of plant development and response to biotic and abiotic stress (Gomez-Roldan et al., 2008; Umehara et al., 2008; Agusti et al., 2011; Kapulnik et al., 2011; Ruyter-Spira et al., 2011; Ha et al., 2014; Decker et al., 2017; Fiorilli et al., 2019).

In *Striga*, the receptors responsible for SL-induced seed germination are evolutionarily derived from a KARRIKIN INSENSITIVE 2 (KAI2) receptor and have arisen through gene duplication and new functionalization replacing the karrikin ligand by SLs (Conn and Nelson, 2016; Xu et al., 2016). Among the several *Striga* SL receptors, *S.* *hermonthica* HYPO-SENSITIVE TO LIGHT (ShHTLs), ShHTL7 is the most sensitive to SLs. It is assumed to play a crucial role in regulating *Striga* seed germination (Tsuchiya et al., 2015).

The dependency of germination on host-derived signals, such as SLs, opens up the possibility of combating root parasitic weeds by applying germination stimulants in the absence of a host (Samejima et al., 2016; Jamil et al., 2018). This “suicidal germination” strategy has been tested recently in field and showed very promising results in alleviating *Striga* infestation (Samejima et al., 2016; Jamil et al., 2018, 2020; Kountche et al., 2019). In addition, the inhibition of SL receptors, that is, DWARF14 (D14) and KAI2-derived receptors, in parasitic and nonparasitic plants by specific SL antagonists is a promising approach for understanding SL perception, combating root parasitic plants, and regulating plant architecture (Ito et al., 2011; Hameed et al., 2018; Nakamura et al., 2019). Recently, we have elucidated the high-resolution atomic structure of ShHTL7 and discovered, by serendipity, that the common detergent Triton X-100 is a noncovalent, specific ligand of this receptor, which competes with the SL ligand and acts as SL antagonist (Hameed et al., 2018). Several tests and modeling confirmed the specificity of Triton X-100 in binding to ShHTL7 among other *Striga* and nonparasitic plant SL receptors (Hameed et al., 2018). In addition, the application of Triton X-100 reduced *Striga* seed germination in vitro without affecting seed germination or rice growth. These results support the idea of using Triton X-100 as a lead compound to design specific and more efficient *Striga* seed germination inhibitors for combating this weed (Hameed et al., 2018). At the same time, Nakamura et al. (2019) reported the development of efficiently covalent inhibitors of D14, taking advantage of the nucleophilicity of the active site Ser residue that mediates SL hydrolysis. This nucleophilicity is generally known for serine hydrolases and enables covalent modification and, hence, covalent inhibition of these enzymes by using reactive electrophiles. Based on studies showing that N-heterocyclic urea structures targeting the serine in the active site are efficient inhibitors of serine hydrolases, Nakamura et al. (2019) prepared a set of triazole urea chemistries and tested their effect on D14 mediated SL perception and signal transduction. Activity tests and structure studies revealed the triazole urea compound KK094 as a potent, covalent inhibitor of the rice D14 (Nakamura et al., 2019). KK094 also inhibited the ShHTL7 receptor, however, with much lower efficiency, and reduced the rate of *Striga* seed germination (Nakamura et al., 2019). In the present work, we developed specific types of ShHTL7 inhibitors, KK023-N1 and -N2, by combining structural elements of Triton X-100 and triazole ureas. The developed compounds, particularly KK023-N1, showed promising activity in blocking ShHTL7 and inhibiting *Striga* seed germination. In contrast to Triton X-100 and KK094, the developed inhibitors did not show any negative effect on rice as host plant. These findings show that KK023-N1 is a promising *Striga*-specific seed germination inhibitor that can be used for combating *Striga* in African agriculture.

## Results

### Design, synthesis, and structure of KK023-N1 and KK023-N2

**Figure 1:**
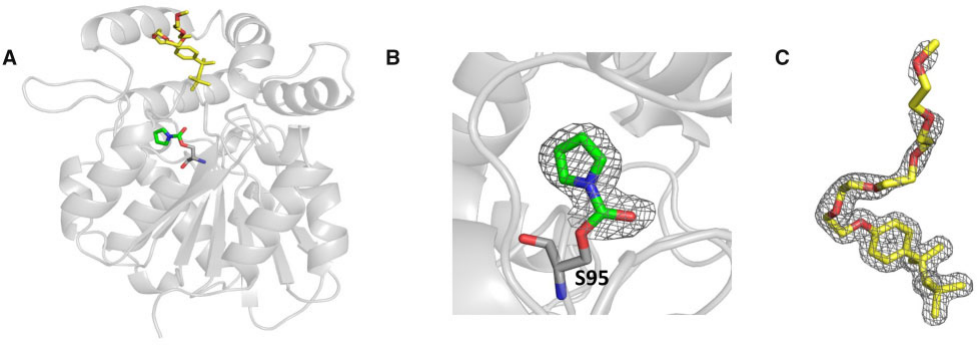
Crystal structure of KK007 and Triton X-100 bound to ShHTL7. A, Crystal structure of KK007 (green stick) and Triton X-100 (yellow stick) bound to ShHTL7 (gray) (B) 2FoFc omit map (gray mesh) shows KK007 (green stick) covalently bound to the active site of ShHTL7_S95_ (gray stick) contoured at 1 σ cutoff (C) 2FoFc omit map (gray mesh) of the bound Triton X-100 (yellow stick) contoured at 1 σ cutoff.

**Figure 2:**
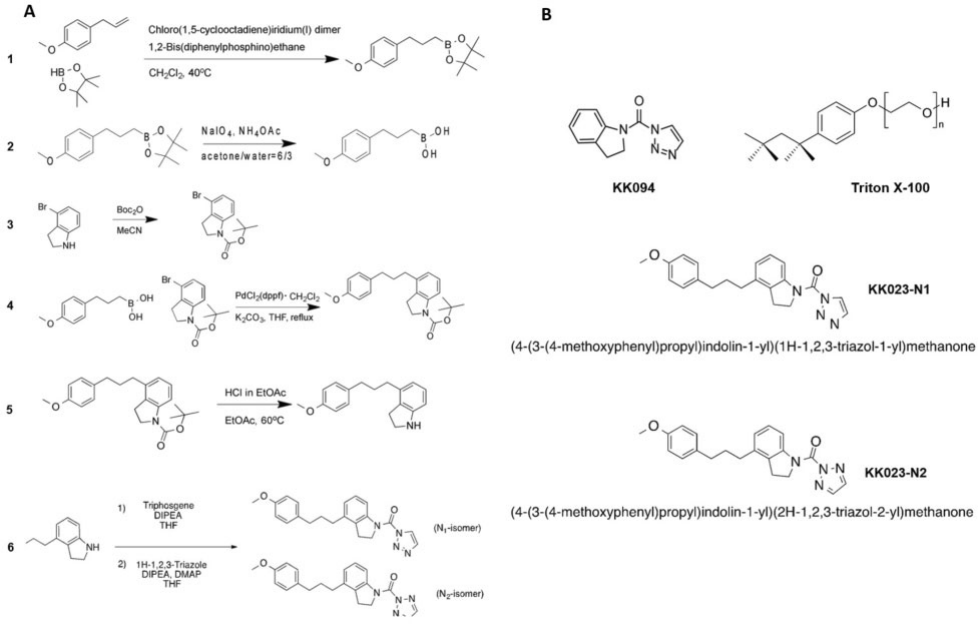
Synthesis of KK023-N1 and KK023-N2. A, The synthesis scheme (steps 1–6) of Triton X-100/1,2,3-triazole urea-based inhibitors. B, Chemical structures of KK094, Triton X-100, KK023-N1, and KK023-N2 isomers.

Previously, the structure of ShHTL7, co-crystallized with Triton X-100, showed that the hydrophobic tail of the ligand, which includes the phenoxy group, interacts with several amino acids in the receptor cavity (Hameed et al., 2018). These interactions provide the basis for the tight binding of Triton X-100 and its blocking/inhibition activity, together with those that take place with the polyethylene repeats at the outer rim of the ShHTL7 binding pocket. Building upon this work, we co-crystallized ShHTL7 with the triazole urea derivative KK007, after incubating them together, and soaked the obtained crystal in 1 mM Triton X-100 solution. Thus, we determined a crystal structure that showed KK007and Triton X-100 bound to ShHTL7, where KK007 was covalently linked to the catalytic Ser95 (Figure  1, A–C; Supplemental Table S1). This structure showed that Triton X-100 left enough space in the active site to accommodate small covalent ligands targeting Ser95, and, hence, suggested that the combination of both moieties might generate efficient and *Striga*-specific inhibitors. Therefore, we attached the alkyl benzene moiety, which corresponds to the hydrophobic tail of Triton X-100 without the methyl groups, to the triazole urea scaffold, which led to the two compounds KK023-N1 and KK023-N2. Attempts to introduce the methyl groups present in Triton X-100 into these hybrid structures were not successful. The synthesis scheme of KK023N1 and -N2 is depicted in Figure  2A, and the chemical structures of KK094, Triton X-100, and the two isomers are in Figure  2B. Nuclear magnetic resonance (NMR) analysis of the two compounds is shown in Supplemental Figure S1.

### KK023-N1 and KK023-N2 are specific inhibitors for ShHTL7

**Figure 3:**
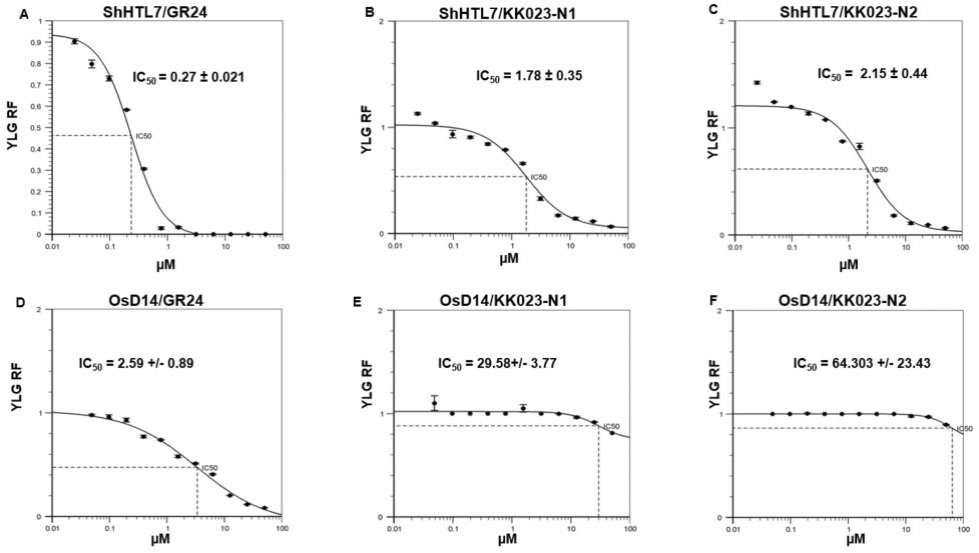
Inhibition of ShHTL7/OsD14 by KK023-N1 and KK023-N2. A, concentration-dependent inhibition of ShHTL7-mediated YLG hydrolysis by (A) synthetic SL GR24 or by (B) KK023-N1 and (C) KK023-N2. Inhibition of OsD14-mediated YLG hydrolysis by (D) GR24, but not with KK023 isomers (E and F). Data are the means ± sd (*n* = 3).

To test the activity of the two compounds, we performed competition assays using Yoshimulactone Green (YLG) as a SL analog that produces a fluorescence signal upon hydrolysis by the SL receptors D14 and ShHTLs (Tsuchiya et al., 2015). ShHTL7 efficiently hydrolyzed YLG, which was detected as an increase of the green fluorescence over time (Supplemental Figure S2A). This activity was completed using SL analog GR24 with an IC_50_ of 0.27 µM (Figure  3A). Then, we tested if KK023-N1 and N2 will competitively inhibit ShHTL7-mediated YLG hydrolysis. As a result, KK023-N1 and N2 inhibited ShHTL7 hydrolysis activity with an IC_50_ of 1.78 and 2.15 µM, respectively (Figure  3, B and C), and this inhibition was concentration-dependent. Under the same conditions, Triton X-100 and KK094 inhibited ShHTL7 activity with an IC_50_ of 0.47 µM and >25 µM, respectively (Supplemental Figure S2, B and C). Furthermore, to check for the specificity of KK023-N1 and KK023-N2, we also evaluated their effect on the rice (*Oryza sativa*) SL receptor D14 (OsD14) receptor. Interestingly, neither of the two isomers could inhibit the hydrolytic activity of OsD14 (Figure  3, E and F), whereas GR24 showed an IC_50_ of 2.59 µM (Figure  3D), indicating the high specificity of the two inhibitors toward the *Striga* ShHTL7 receptor. Likewise, we tested if KK023-N1, the more potent inhibitor, inhibits the *Striga* KAI2-derived SL receptor ShHTL5 that hydrolyzes YLG (Supplemental Figure S2D). We observed only weak inhibition of the hydrolysis activity of purified ShHTL5, albeit the high sequence similarity to ShHTL7 (Hameed et al., 2018; Xu et al., 2018). Indeed, the application of KK023-N1 at a 50 µM concentration led to only a 20% reduction of ShHTL5 hydrolysis activity, indicating an IC_50_ > 50 µM (Supplemental Figure S2E).

**Figure 4:**
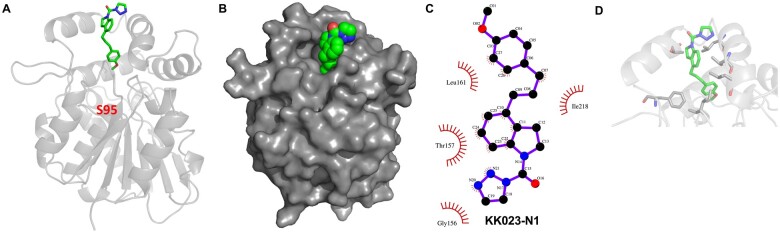
Docking of KK023-N1 to ShHTL7. A, Cartoon representation of KK023-N1 (green stick) docked to ShHTL7 (gray) (B) Surface representation of KK023-N1 (green sphere) docked to ShHTL7 (gray) (C) Ligplot shows residual contacts of ShHTL7 to docked KK023-N1 (D) Contact residues (gray) of ShHTL7 to docked KK023-N1 (green stick).

The specificity of KK023-N1 toward ShHTL7 could be seen from in silico docking of KK023-N1 to ShHTL7, where it could bind noncovalently in the lowest-energy model between the helices α3–α4 at the entrance of the ShHTL7 binding pocket, primarily through hydrophobic interactions with Thr157 and Ile161 (Figure  4, A–D). This model provides a rationale for the loss of binding by KK023-N1 toward OsD14 and ShHTL5 due to the presence of bulky amino acids such as Tyr and Trp in their ligand cavity, which hinders the binding of KK023-N1.

### KK023-N1 decreased binding affinity between ShHTL7 and ShMAX2-CTH and increased ShHTL7 thermal stability

**Figure 5:**
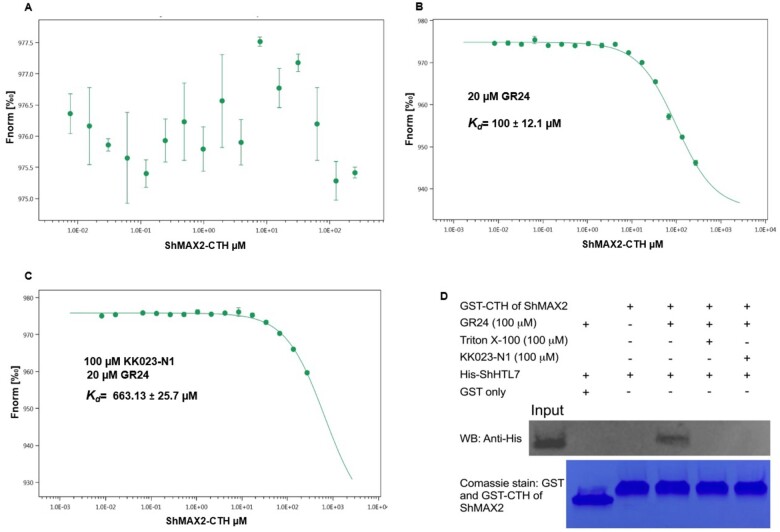
Determination of *K*_d_ by MST for the interaction between ShHTL7 and ShMAX2-CTH. A, The binding affinity between 20 nM of labeled ShHTL7 and ShMAX2-CTH was analyzed in the buffer and no direct interaction was detected between the two proteins in the absence of GR24. B, Addition of 20 µM of GR24 appeared very active to induce the ShHTL7–ShMAX2–CTH interaction. C, The binding affinity shifted upon the addition of KK023-N1 with a dissociation constant *K_d_* = 663.13 ± 25.7 µM, indicating that KK023-N1 is blocking the GR24-dependent interaction between ShHTL7–ShMAX2–CTH. Data are the means ± sd (*n* = 3). D, GST pull down of His-ShHTL7 by CTH of ShMAX2 in the presence of GR24, Triton X-100, and KK023-N1.

We then investigated whether KK023-N1 interferes with the binding of ShHTL7 to the downstream signal transduction component *S.* *hermonthica* MORE AXILLARY BRANCHING 2 (ShMAX2). For this purpose, we first investigated if the C-terminal helix of ShMAX2 (ShMAX2-CTH) is sufficient for binding to ShHTL7 in the presence of the SL analog GR24, based on the known fact that the C-terminal helix of D3 is sufficient for interaction with D14 in the presence of GR24 (Shabek et al., 2018). Using Microscale thermophoresis (MST), we found that GR24 facilitates binding between ShHTL7 and ShMAX2-CTH with a *K*_d_ value of ∼100 µM, and there was no binding in the absence of GR24 (Figure  5, A and B). To determine whether KK023-N1 inhibits this interaction, we performed the measurement using ShHTL7 pre-incubated with KK023N1, which resulted in a substantial reduction in the binding affinity (*K*_d_ = 663 µM), indicating that KK023-N1 blocks the ShHTL7 binding pocket and thus prevents the SL-dependent interaction with ShMAX2 (Figure  5C). Interaction between ShHTL7 and CTH of MAX2 in the presence of GR24 was further confirmed by GST pull-down. We found GST tagged CTH of ShMAX2 could pull down His-ShHTL7 in the presence of GR24, and KK023-N1 could inhibit such interaction; also, we have used Triton X-100 as control which confirmed that KK023-N1 is sufficient to inhibit ShHTL7 activity (Figure  5D).

**Figure 6:**
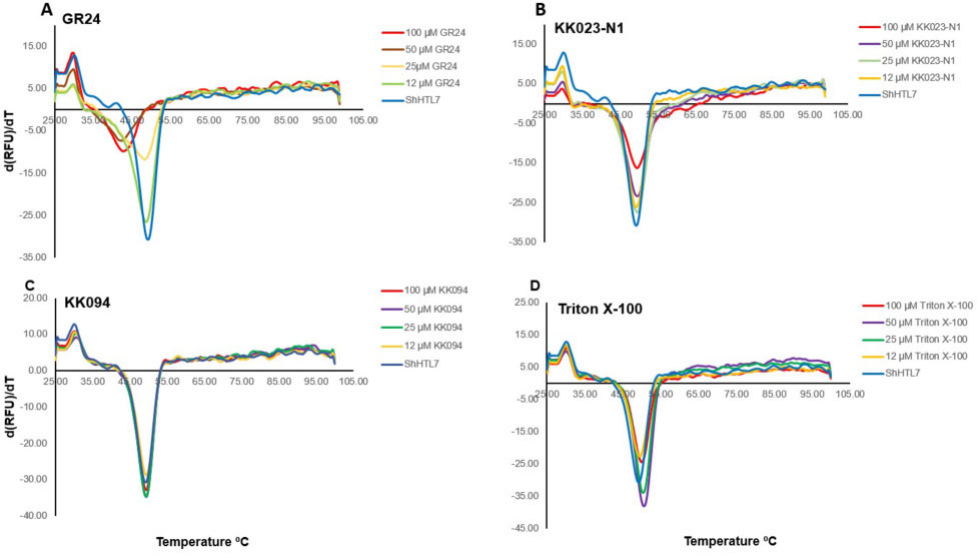
Evaluation of ShHTL7 thermal stability and interaction with KK023-N1 and GR24 using differential scanning 945 fluorimetry (DSF). Melting temperature curve for ShHTL7 at varying concentrations of (A) KK023-N1 or (B) GR24 is assessed by DSF. Protein unfolding was detected by the changes of fluorescence of SYPRO Orange dye. The inverse peak minima inferred to the melting temperature. Data are means (*n* = 3) measured in parallel.

To further confirm KK023-N1 binding to ShHTL7, we measured the thermal stability of the receptor, using GR24 as a comparison. The melting temperature of ShHTL7 decreased from 50°C to 43°C in the presence of high concentrations of GR24 (Figure  6A). In contrast, incubation with KK023-N1 did not affect the melting temperature of ShHTL7. Interestingly, the application of high KK023-N1 concentrations decreased the fluorescence of the SYPRO orange indicating an interaction and binding of this inhibitor to the protein in a concentration-dependent manner. Triton X-100 also showed the same pattern as observed for KK023-N1 with a reduction in the peak fluorescence, while incubation with KK094 did not change the fluorescence peak (Figure  6, B–D).

### KK023-N1 and KK023-N2 inhibited *Striga* seed germination

**Figure 7:**
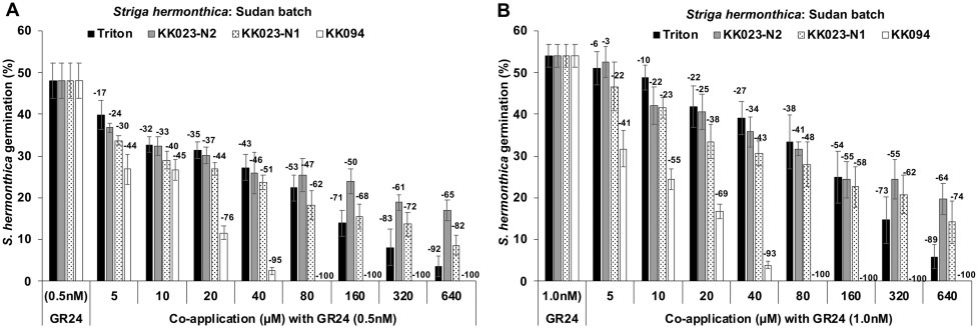
Effect of Triton X-100, KK023-N1, KK023-N2, and KK094 on *S. hermonthica* seed germination. Preconditioned *Striga* seeds (∼50–100 per disc) were first pretreated with the indicated concentrations of inhibitors for 24 h, and then mixture of each inhibitor at the indicated concentrations along with GR24 at (A) 0.5 nM and (B) 1 nM was applied for another 24 h. Germinated and nongerminated seeds were counted from each disc to calculate percentage germination. Data are means ± se (*n* = 6 discs). The negative value on top of each bar is the percentage reduction of *Striga* seed germination over control.

The in vitro studies with the receptor ShHTL7 indicated that KK023-N1 and KK023-N2 could inhibit *Striga* seed germination. To test this possibility, we applied KK023-N1, -N2, Triton X-100, and KK094 at varying concentrations along with GR24 (at 0.5 or 1.0 nM) on preconditioned *Striga* seeds (collected from Sudan). Both KK023-N1 and -N2 showed inhibition of *Striga* seed germination in a concentration-dependent manner. In general, KK023-N1 appeared more effective than KK023-N2 (Figure  7, A and B). Application of KK023-N1 between 20 and 40 µM concentrations led to around 22%–51% reduction compared to the GR24 treatment (at 0.5 or 1.0 nM). KK094 showed complete inhibition at 80 µM, and the same trend has been reported previously (Nakamura et al., 2019). However, Triton X-100 appeared weaker than KK023, showing a 20%–35% reduction in *Striga* seed germination with a 20 µM concentration. In another study, we also tested the inhibitory activity of KK023-N1 and -N2 on *Striga* seeds obtained from Kenya. We found considerable reduction (28%–100%, depending on applied concentrations) in *Striga* seed germination by both inhibitors compared to control (Supplemental Figure S3).

### KK023-N1 and KK023-N2 did not impact rice seeds growth or tillering

**Figure 8:**
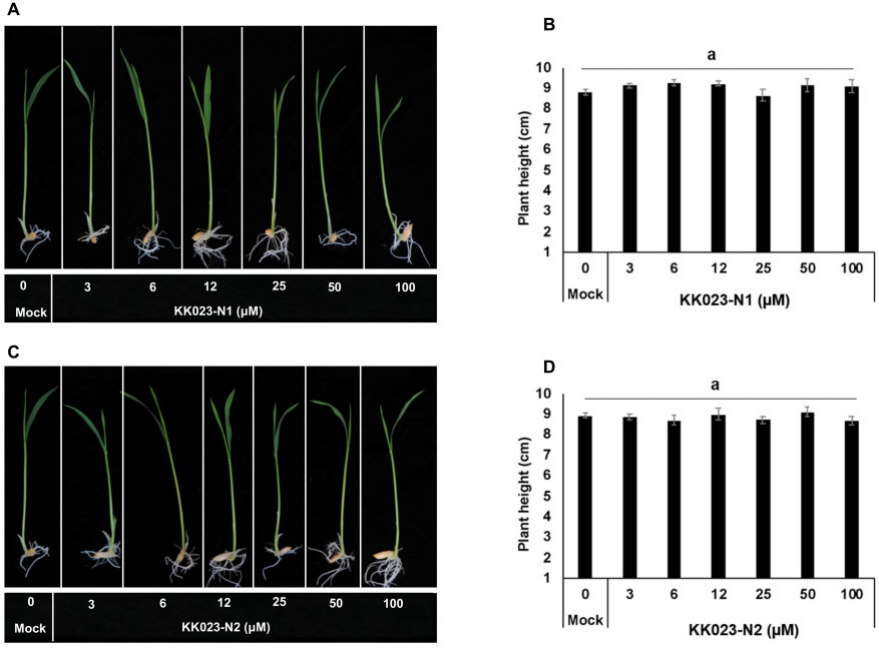
Effect of KK023-N1 and KK023-N2 on rice seeds germination and seedlings growth. Rice seeds were germinated in 24-well plate in 0.5 MS medium along with various concentrations of (A and B) KK023-N1 and (C and D) KK023-N2. The growth of rice seedling was assessed for 1 week and no effect on plant height was observed under all treatments. Data are means ± se (*n* = 6). Means not sharing a letter in common differ significantly at *P*_0.05_ (analysis of variance (ANOVA) test).

In addition, we have assessed KK023-N1 and -N2 impact on rice seed germination and seedling development. For this purpose, we applied various concentrations (up to 100 µM) of both compounds on imbibed rice seeds and allowed them to grow for 2 weeks. We did not detect any effect on seed germination (Figure  8, A and B). Furthermore, rice seedlings treated with KK023-N1 or KK023-N2 did not show any significant phenotypic difference to the control seedlings.

**Figure 9:**
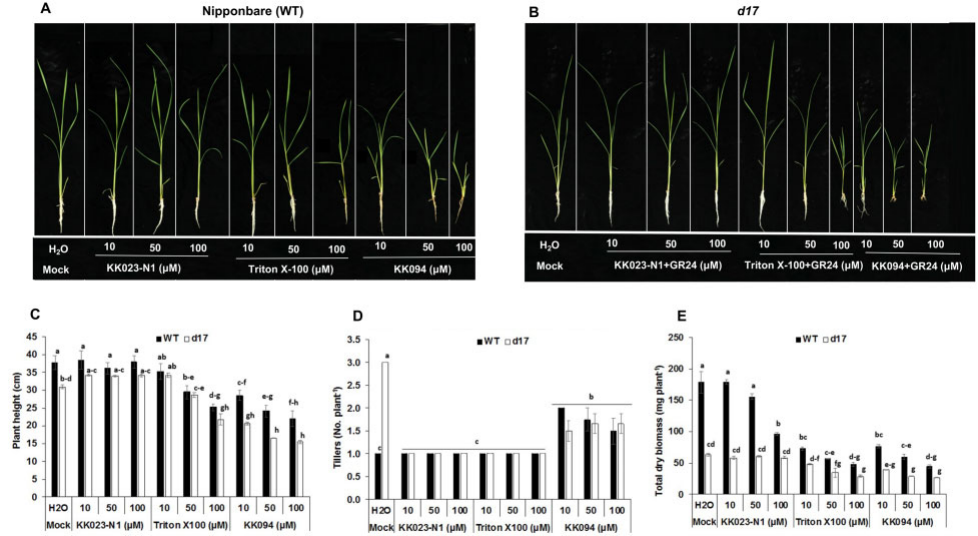
Effect of KK023-N1, Triton X-100, and KK094 on growth of rice seedlings in Nipponbare (WT) and *d17* under hydroponics conditions. Phenotype of (A) Nipponbare (WT) and (B) *d17* rice seedlings treated with varying concentrations of each compound (±GR24 in *d17*) for 2 weeks. C, Rice seedling height in response to KK023-N1, Triton X-100, and KK094 application. All the treatments of KK023-N1 in WT and in *d17* (±1 µM GR24) did not affect plant height while high concentrations of Triton X-100 led to dwarfness (D). Effect of KK023-N1, Triton X-100, and KK094 application on rice tillering. Both KK023-N1 and Triton X-100 had no effect on rice tillering in WT (1 per plant) while application of KK094 in WT and *d17* (±1 µM GR24) led to production of second tiller. E, Total dry biomass of rice as affected by application of KK023-N1, Triton X-100, and KK094. Total dry biomass of rice (root + shoot) remained unaffected by lower doses of KK023-N1 (±1 µM GR24 in *d17*) while high doses of KK023-N1 in WT and all doses of Triton X-100 and KK094 (Both WT and *d17*) caused reduction in total dry biomass. Data are means ± se (*n* = 4). Means not sharing a letter in common differ significantly at *P*_0.05_ (ANOVA test).

Previously, Nakamura et al. (2019) demonstrated that KK094 inhibits SL perception by D14 and induces the emergence of second tillers by interrupting the SL signaling pathway. This raises the question of whether our compounds also interfere with D14 and promote bud outgrowth in rice. To test this hypothesis and the specificity of KK023 isomers toward *Striga* weeds, we firstly applied KK023-N1, Triton X-100, and KK094 (at 10, 50, and 100 µM) to wild-type (WT) plants. We did not observe any significant negative impact of all concentrations of KK023-N1 on plant height and rice tillering in WT, except at 100 µM concentration that led to a reduction in biomass (Figure  9, A, C, and E). However, the application of Triton X-100 at high concentrations caused a reduction in plant height and dry biomass. The negative impact of Triton X-100 on dry biomass was detected already at a concentration of 10 µM, Both KK023 and Triton X-100 did not affect tillering in WT rice. In contrast, the application of KK094 led to a significant increase in rice tillering and a reduction in plant height and dry biomass in WT plants, demonstrating the negative impact of this compound on the growth of the host plant rice (Figure  9, A, C, and E). To further validate the effect of KK023-N1, Triton X-100, and KK094 on SL perception and ultimately on rice tillering phenotype, we applied them in combination with 1.0 µM GR24 to seedlings of the SL deficient rice mutant *d17* (Figure  9B). KK023-N1 and Triton X-100 did not block the GR24 effect on rice tillers (Figure  9D), which indicated that D14 does not bind these two compounds, and their SL antagonistic activity is specific to *Striga* seed germination, whereas KK094 blocked D14, resulting in increased tillering phenotype (Figure  9D).

### KK023-N1 reduced *Striga* emergence in infested rice under greenhouse conditions

**Figure 10:**
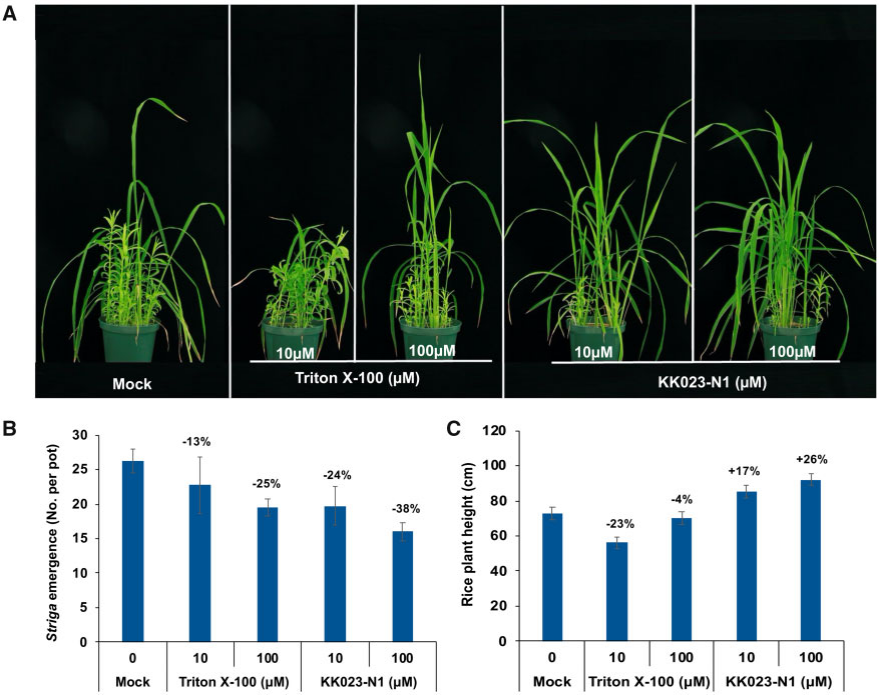
Effect of KK023-N1 and Triton X-100 on *Striga* emergence in rice under greenhouse conditions. Formulated KK023-N1 or Triton X-100 was applied at two concentrations (10 and 100 µM), twice per week up to 4 weeks period. The emergence of *Striga* plants was recorded after 10 weeks of rice planting. A, *Striga* infestation in treated and nontreated rice plants. B, Number of *Striga* emergence in response to KK023-N1 and Triton X-100 application. The negative values on the top of each bar represent reduction in *Striga* emergence (in percentage) as compared to mock (C) rice plant height in treated and nontreated pots. Data are means ± se (*n* = 4).

Next, we evaluated the effect of KK023-N1 on *Striga* emergence in artificially infested pots planted with rice as a host crop (Figure  10A). *Striga* emergence was recorded 10 weeks after rice planting. On an average, we observed maximum *Striga* emergence (26 per pot) in the mock-treated pots, while pots treated with KK023-N1 at 10 and 100 µM, showed an average of 20 and 16 *Striga* plants per pot, respectively, which corresponds to a reduction of 24%–38% in *Striga* emergence (Figure  10B). Also, the Triton X-100 treated pots (at 10 and 100 µM) exhibited a reduction in the *Striga* emergence (∼13%–25%). However, the number of emerged *Striga* plants in Triton X-100 treated pots was higher as compared to the KK023-N1 treatment. Plant height was recorded at the end of the study, and application of KK023-N1 showed even a positive impact on plant height, as compared to mock and Triton X-100 treatments (Figure  10C).

## Discussion

The control of the noxious parasitic weed *Striga* has become a significant challenge for global food production, particularly in sub-Saharan Africa, despite the efforts deployed to combat it over the last decades. The persistence of the *Striga* problem compels scientists to develop alternatives and more efficient methods for combating this weed. Suicidal germination, a strategy that takes advantage of the dependency of parasitic seeds on host-released germination stimulants to diminish accumulated seed banks, has been recently demonstrated to be efficient in alleviating *Striga* infestation (Uraguchi et al., 2018; Kountche et al., 2019). However, this strategy can only be applied in the absence of the host, which means that farmers cannot plant their fields with cereals when cleaning off their soils. The development of specific inhibitors of *Striga* seed germination offers a complementary approach for combating *Striga*, which can be applied in parallel to host crop cultivation. In this work, we combined the structure of Triton X-100 and the scaffold of triazole urea compounds (KK series; Nakamura et al., 2019) to develop chemistries with high efficiency in blocking *Striga* seed germination, and which are suitable for field application. Our combinatory approach led to the two isomers, KK023-N1 and KK023-N2, as inhibitor candidates.

We first characterized the inhibitory activity of the two candidates using in vitro YLG competition assays with purified ShHTL7 and D14 protein. The fluorescent substrate YLG has been widely used to measure the hydrolysis activity of SL receptors. The IC_50_ determined for ShHTL7 in the presence of KK023-N1 or KK023-N2 were 1.78 and 2.15 µM, respectively, which are much lower than that observed for OsD14. Additionally, both KK023-N1 and KK023-N2 did not influence the YLG hydrolysis by ShHTL5, despite the high sequence similarity with ShHTL7. Structure analysis revealed that ShHTL7 possesses a large ligand-binding pocket among other SL receptors in parasitic and nonparasitic plants (Xu et al., 2018). The increase in the pocket volume is due to the movement of the αD1 helix outward from the entrance of the pocket. Also, the ShHTL7 binding pocket is composed of nonbulky residues, compared to OsD14 and ShHTL5, and this explained the basis for the ligand selectivity (Hameed et al., 2018; Xu et al., 2018). The presence of the triazole urea scaffold in KK023-N1 and -N2 makes covalent binding to the active site serine in ShHTL7 likely, as shown for KK007 and the D14 inhibitor KK094 (Nakamura et al., 2019). However, our YLG competition assay (Figure  3, A–C) indicated that KK023-N1 is outcompeted by GR24, suggesting the binding to be noncovalent. Indeed, MALDI-TOF and LC-MS analysis of protein and peptides (Supplemental Method S1) obtained by digesting ShHTL7 upon incubation with KK023-N1 did not indicate covalent modification to the receptor (Supplemental Figure S4, A and B). Our docking model of KK023-N1 bound to ShHTL7 also suggests that the compound does not reach deep enough in the ShHTL7 binding pocket to use its potential for covalent linkage with the catalytic Ser95 (Figure  4A). Therefore, KK023-N1 might interact noncovalently with ShHTL7, as previously shown for Triton X-100 (Hameed et al., 2018). Soporidine (SOP) (Holbrook-Smith et al., 2016; Yoneyama, 2016), a previously reported *Striga* seed germination inhibitor, was shown to competitively inhibit ShHTL7-mediated YLG hydrolysis with an IC_50_ of 12.5 µM. This value is around six-fold higher than our inhibitors KK023-N1 and KK023-N2, indicating a higher efficiency of our compounds in blocking ShHTL7, the most sensitive SL receptor in *Striga*. Our YLG hydrolysis assay showed that KK023-N1 and KK023 could competitively prevent YLG from being hydrolyzed by ShHTL7, indicating that they are very likely bound to close proximity of the binding pocket and thus prevent YLG from being bound to the ShHTL7 active site. Moreover, our docking model also had the lowest binding energy possible when the compound is bound to the ShHTL7 pocket rather than any other site.

In SL signal transduction, the receptor D14 interacts with D3 (MAX2 homolog) in a SL-dependent manner (Waters et al., 2012). Upon binding to SL, D14 undergoes a conformational change that facilitates the interaction with MAX2 (Jiang et al., 2013; Nakamura et al., 2013; Yao et al., 2018; Yoneyama et al., 2019; Wang et al., 2021). Based on the high sequence similarity between ShMAX2 and D3 receptor anchoring site, ShHTL7 binding to ShMAX2 was shown to be also SL-dependent (Yao et al., 2017; Hameed et al., 2018), and this interaction was precluded by the presence of the inhibitor Triton X-100 (Hameed et al., 2018). D3-CTH of rice was already shown to be sufficient for triggering this interaction (Shabek et al., 2018). Using MST, we showed in our study that ShHTL7 binding to ShMAX-CTH is SL-dependent, suggesting that ShHTL7 undergoes a conformational rearrangement upon SL binding, which establishes the association with ShMAX2-CTH. The *K*_d_ value for this interaction indicated modest binding affinity with 100 µM, but this could be because we only used a shorter purified CTH of ShMAX2 fused to GST. Also, taking into consideration that we might be missing other sites of ShMAX2 involved in ShHTL7–SHMAX2 complex formation. However, our study aimed to validate the association of this peptide. We have shown that it binds in a SL-dependent manner and that the addition of KK023-N1 perturbed SL binding caused a substantial, six-fold reduction in the affinity of ShHTL7 toward ShMAX2-CTH (Figure  5, A–C). GST pull-down study further confirmed that KK023-N1 could inhibit ShHTL7 activity and thus prevents it from hydrolyzing its substrate and binding to the downstream regulator (Figure  5D). The effect of KK023-N1 on ShHTL7–ShMAX2-CTH interaction clearly demonstrates that the occupation of ShHTL7 binding pocket by the inhibitor prevents the occurrence of the structural changes required to anchor ShMAX2 and antagonist the effect of GR24, the synthetic SL. Moreover, we have shown using differential scanning fluorimetry (DSF) that the occupation of the receptor cavity by KK023-N1 stabilized the ShHTL7 structure and reduced its susceptibility to thermal denaturation, decreasing the amount of the hydrophobic peptides are required for the binding with SYPRO orange dye. Likewise, Triton X-100 in DSF assay showed the same trend, and with higher concentrations, the peak depth was lower than that for ShHTL7. While GR24 binding decreased the ShHTL7 melting temperature by 7°C, the interaction with KK094 did not change the melting peaks’ pattern, which is consistent with the IC_50_ obtained from the YLG study (<25 µM; Supplemental Figure S2C). Based on all studies that were conducted here and in (Nakamura et al., 2019), KK094 weakly inhibited ShHTL7 compared with Triton X-100 and KK023 isomers.

Following the in vitro tests with purified receptors, we performed *Striga* seed germination inhibition studies (in the presence of germination stimulant) under lab conditions and detected a reasonable activity with both compounds. Application of KK023-N1 (at 20–40 µM) along with 0.5 or 1 nM GR24 reduces the germination of *Striga* seeds by around 50%, compared to the treatment with GR24 alone, while KK023-N2 at the same concentrations caused around 42% decrease in germination (Figure  7, A and B). KK094 showed50% inhibition of *Striga* seeds germination at 20 µM concentration and led to complete inhibition (100%) at 100 µM. Application of Triton X-100 (at 20–40 µM) showed a 31% reduction in *Striga* seed germination, which is below the values reported before (Hameed et al., 2018). This difference in Triton X-100 activity might be attributed to the difference in *Striga* seeds batches used in each bioassay, that is, seeds used in this study showed higher germination activity than those used by Hameed et al. (2018). Moreover, the value obtained for the inhibitory effect of Triton X-100 on *Striga* seed was the outcome of the median of variant GR24 concentrations (1, 0.5, 0.25, and 0.125 nM) indicated by Hameed et al. (2018).

We also assessed the effect of KK023-N1 and -N2 on the growth of rice as a host plant and did not observe any negative impact on plant height of the treated seedlings up to 100 µM (Figure  8, A and B). In another study, the application of KK023-N1 at 10, 50, and 100 µM did not show any effect on plant height of WT and SL deficient *d17* (±GR24) rice seedlings (Figure  9, A–E). Application of KK023-N1 at 100 µM showed minor reduction in the biomass of rice seedlings, compared to KK094 or Triton X-100. Moreover, we did not detect a negative impact of KK023-N1 on rice in our greenhouse study. Our findings also show that Triton X-100 and KK023 isomers are ShHTL7 specific and selective inhibitors and did not bind to OsD14 in vitro. Here, we have provided strong evidence that none of these inhibitors (KK023-N1, Triton X-100) restore the tillering phenotype and do not interfere with SL perception in the host plants. In addition, the application of KK023-N1 and -N2 on other *Striga* ecotypes (Kenya) reduced their germination rate. Consequently, these outcomes confirm the possible use of the proposed inhibitors (KK023-N1 and -N2) against *Striga* in host plant presence. Based on the results of YLG competition assays, *Striga* germination bioassays, and *Striga* pot study, KK023-N1 appeared more active than KK023-N2 with a 38% reduction in *Striga* emergence under greenhouse conditions (Figure  10, A and B). KK094 expressed a high inhibitory activity on *Striga* seeds germination compared to KK023 and Triton X-100. Yet, the negative effect of KK094 application on host plants (hydroponic study Figure  9) has to be overcome, and further modifications have to be done in order to use this compound as a specific inhibitor for *Striga* seed germination.

Inhibiting the *Striga* seed germination by blocking the perception of the SL germination stimulant could be a very efficient approach for combating *Striga*. Our hybrid inhibitors, particularly KK023-N1, showed potential inhibition in *Striga* seed germination and are characterized by their specificity and low impact on the host. These encouraging findings indicate the potential of these compounds for application in combating *Striga* and make them excellent lead compounds for developing further *Striga*-Specific herbicides that can be applied in the presence of a host. For practical field application, we are planning field trials in infested regions in Burkina Faso in the future.

## Materials and methods

### Chemicals and formulation

The standard SL analog GR24 was kindly provided by Prof. Binne Zwanenburg, Radboud University, Netherlands. GR24 was applied as a racemic mixture (*rac*-GR24) of two stereoisomers that differ in the configuration of C2′ atom (2′*R* and 2′*S* configuration). Other chemicals used to prepare half-strength Hoagland’s nutrient solutions, 2-(N-morpholino) ethane sulfonic acid (MES), and dimethyl sulfoxide (DMSO) were purchased from different suppliers. Seeds of *S.* *hermonthica* were collected from an infested Sorghum (*Sorghum bicolor*) field near Wad Medani, Sudan, and provided by Prof. Abdel Gabar Babiker. *Striga* seeds collected from Maize infested field from Kenya, were provided by Prof. Steven Runo (Kenyatta University, Kenya). Seeds of the *Striga* susceptible rice *cv* IAC 165 were kindly provided by Dr Jonne Rodenburg, Africa Rice, Tanzania.

### Synthesis of 2-[3-(4-methoxyphenyl)propyl]-4,4,5,5-tetramethyl-1,3,2 dioxaborolane

A mixture of chloro(1,5-cyclooctadiene)iridium(I) dimer (90.6 mg, 0.135 mmol) and 1,2 Bis(diphenylphosphino)ethane (107.5 mg, 0.270 mmol) was stirred for 5 min at 40°C under argon atmosphere in dichloromethane (20 mL). Then 1-allyl-4-methoxybenzene (2.0 g, 13.49 mmol) and 4,4,5,5-tetramethyl-[1,3,2]dioxaborolane (2.1 g, 16.19 mmol) were added to the reaction mixture and stirred for 48 h at room temperature. After completion of the reaction, the mixture was passed through a silica gel short column to remove the unsolved material and then concentrated in vacuo. The residue was purified with silica gel (WakosilC-300HG) column chromatography (*n*-hexane:ethyl acetate) to give the target compound in 93% yield.

### Synthesis of B*-*[3-(4-methoxyphenyl)propyl]-boronic acid

A mixture of 2-[3-(4-methoxyphenyl)propyl]-4,4,5,5-tetramethyl-1,3,2-dioxaborolane (1.0 g, 3.62 mmol), acetone (43 mL), distilled water (21.7 mL), sodium periodate (2.32 g, 10.86 mmol), and ammonium acetate (837 mg, 10.86 mmol) was stirred for overnight at room temperature in a round bottom flask. The reaction mixture was then diluted with ethyl acetate (50 mL), washed with brine (50 mL). The aqueous phase was then extracted twice with ethyl acetate (30 mL). The combined organic phase was dried with sodium sulfate and concentrated in vacuo. The residue was purified with silica gel (WakosilC-300HG) column chromatography (n-hexane:ethyl acetate) to give the target compound in 72% yield.

### Synthesis of the 4-Bromo-2,3-dihydro-indole- 1-carboxylic acid tert-butyl ester

A mixture of 4-Bromo-2,3-dihydro-1H-indole (1.0 g, 5.10 mmol), acetonitrile (10.2 mL), and Boc_2_O (1.34 g, 6.12 mmol) was stirred overnight at room temperature in a round bottom flask. The reaction mixture was then diluted with organic solvent (50 mL of *n-*hexane:ethyl acetate) and washed with brine (50 mL). The combined organic phase was dried with sodium sulfate and concentrated in vacuo. The residue was purified with silica gel (WakosilC-300HG) column chromatography (*n*-hexane:ethyl acetate) to give the target compound in 98% yield.

### Synthesis of *tert*-butyl 4-(3-(4-methoxyphenyl)propyl)indoline-1-carboxylate

A mixture of *B*-[3-(4-methoxyphenyl)propyl]boronic acid（504.1 mg, 1.83 mmol), 2,3 dihydroindole-1-carboxylic acid tert-butyl ester (542.4 mg, 1.83 mmol), tetrahydrofuran (THF) (18.3 mL), potassium carbonate (759.7 mg, 5.49 mmol) and 1,1′-bis(diphenylphosphino)ferrocene- palladium(II)dichloride dichloromethane complex (137.1 mg, 0.165 mmol) was refluxed for 18 h. Then the reaction mixture was diluted with ethyl acetate (30 mL) and washed successively with water (50 mL) and brine (50 mL). The combined organic phase was dried with sodium sulfate and concentrated in vacuo. The residue was purified with silica gel (WakosilC-300HG) short column chromatography to remove the unsolved material and then concentrated in vacuo. The resulted oil was used in the next step without further purification.

### Synthesis of 4-(3-(4-methoxyphenyl)propyl)indoline

The mixture of *tert*-butyl 4-(3-(4-methoxyphenyl)propyl)indoline-1-carboxylate (1.83 mmol), ethyl acetate (18.0 mL) and hydrogen chloride ethyl acetate solution (4 mol/L, 1.0 mL) was stirred for 1 h at 60°C. The reaction mixture was concentrated in vacuo and purified with silica gel (WakosilC-300HG) column chromatography (*n*-hexane:ethyl acetate) to give the target compound in 67% yield.

### Synthesis of Triton/Triazole urea hybrids

A solution of 4-(3-(4-methoxyphenyl)propyl)indoline (252 mg, 0.94 mmol) and *N*,*N*diisopropylethylamine (DIPEA; 365 mg, 2.83 mmol) in THF (3 mL) was added dropwise to a triphosgene (140 mg, 0.47 mmol) solution in THF (5 mL) under ice-cooling. After stirring with cooling for 20 min, iced water (10 mL) and ethyl acetate (10 mL) were added to the reaction mixture, and extraction was carried out. The combined organic phase was washed with iced water, dried with anhydrous sodium sulfate, and concentrated in vacuo. The residue was dissolved in THF (5 mL). To this solution, 1*H*-1,2,3-Triazole (78 mg, 1.13 mmol), DIPEA (365 mg, 2.28 mmol), and DMAP (trace) were added at room temperature. After stirring at room temperature overnight, the mixture was diluted with ethyl acetate and washed with water. The organic phase was dried with anhydrous sodium sulfate, and the solvent was concentrated in vacuo. The residue was purified by silica gel column chromatography (*n*-hexane:ethyl acetate = 4:1–3:2) to give the products of N1-isomer (141 mg, 41%) as a white solid and N2-isomer (72 mg, 21%) as a white solid.

### Cloning, expression, and purification of ShHTL7, ShHTL5, OsD14, and ShMAX2CTH recombinant proteins

cDNAs were cloned and proteins were purified as described earlier in Hameed et al. (2018). Briefly, ShHTL7, OsD14, and ShHTL5 were amplified using gene-specific primers (Supplemental Table S2) and obtained polymerase chain reaction (PCR) fragments were digested with BamHI and XhoI for ShHTL7 and OsD14 or with EcoRI and XhoI for ShHTL5 and cloned into pGEX-6P-1 (GE Healthcare, Chicago, IL, USA) vector. For His-tagged ShHTL7, the cDNA was cloned between BamHI and NotI site to pQlinkH expression vector. Recombinant plasmids were transformed into *Escherichia* *coli* BL21 (DE3) and the Expression of the recombinant proteins was induced by the addition of 150-µM isopropyl b-d-1-thiogalactopyranoside, and induced cultures were incubated for 18 h at 16°C. Cells were harvested by centrifugation then resuspended in lysis buffer (50 mM Tris–HCl pH 8.0, 200 mM NaCl, 2 mM DTT, 0.5% (v/v) Tween-20) followed by sonication for 10 min with 2 s ON and 1 s OFF at amplitude 40% on ice. The Cleared lysate was loaded into the Glutathione Sepharose 4B resins and allowed to bind at 4°C for 2 h. For eluting the proteins, ShHTL7 and ShHTL5 were eluted by cleavage of GST tag using PreScission Protease (GE Healthcare), and OsD14 was eluted by adding 20 mM reduced glutathione (Sigma-Aldrich, St Louis, MO, USA) with adjusting the pH to 8.0. ShMAX2-CTH coding sequence was extracted using sequence alignment with reported CTH of OsD3 (Shabek et al., 2018) and synthesized as gblock (IDT) (Supplemental Table S2). ShMAX2-CTH was digested with EcoRI and XhoI and cloned into pGEX-6P-1, using CEPC cloning procedure (Quan and Tian, 2011). Expression and purification of CTH were performed by adopting the same procedure as mentioned above. For His tagged ShHTL7 purification, it was lysed in the buffer (50-mM Tris–HCl pH 8.0, 200 mM NaCl, 1 mM DTT, 0.5% Tween-20) and allowed to bind Ni-NTA agarose beads (Qiagen, Hilden, Germany) for 1 h at 4°C. Then washed three times with 30 mM Imidazole for 15 min each wash and eluted with 250 mM Imidazole and then passed through gel filtration as above for other proteins.

### Crystal structure determination of KK007 and Triton X-100 bound to ShHTL7

Purified ShHTL7 was incubated with an excess molar of KK007 and crystallized in buffer containing 20% (w/v) PEG 8000, 100 mM MES/Sodium hydroxide pH 6.0, and 200-mM calcium acetate under vapor diffusion method. Thus, the obtained crystal was soaked in crystallization buffer containing 1 mM Triton X-100. The crystals were flash cooled in liquid nitrogen with 25% (v/v) glycerol as cryo-protectant. Data were collected at 100 K at the beamlines Proxima 1 and Proxima 2A at the SOLEIL Synchrotron (France) (proposal numbers 20161236, 20170193). The data were processed in XDS. The structure was determined by molecular replacement using MoRDa with the ShHTL7 structure (PDB 5Z89) as a search model. The structures were manually investigated using Coot and refined using Phenix Refine (Supplemental Table S1). Co-ordinates for the crystal structure was deposited in PDB with accession code 7C8L.

### YLG hydrolysis assays

About 0.33 µM of ShHTL7 protein was used to conduct in vitro hydrolysis of YLG (1 µM) as a substrate in a total volume of 100 µL of phosphate-buffered saline (1× PBS) containing 0.1% DMSO in 96-well black plate (Greiner Kremsmünster, Austria; Tsuchiya et al., 2015). The hydrolysis of YLG over time and changes in the fluorescence intensity was measured by SpectraMax i3 (Molecular Devices, San Jose, CA, USA) using an excitation wavelength of 480 nm and detection wavelength of 520 nm and recorded at 10 min intervals for 2 h. YLG in 1× PBS without protein was used as a control. For competitive inhibition of YLG hydrolysis in the presence of the compounds (KK023-N1 or -N2, Triton X-100, and KK094), proteins (ShHTL7, OsD14, ShHTL5, at 0.33 µM final concentration) were co-incubated with different concentrations ranges (20 nM to 50 µM) of both inhibitors for 1 h at room temperature, then 1 µM of YLG was added and incubated for another 1 h. Measurement of the fluorescence was conducted in SpectraMax i3 (Molecular Devices) using the above-mentioned method. The relative fluorescence unit was calculated by subtracting the auto-fluorescence of YLG in the buffer from the fluorescence values of YLG in the presence of a protein. The inhibitory curve and IC_50_ were determined using https://www.aatbio.com/tools/ic50-calculator website.

### MST measurement of the binding affinity of ShHTL7 to ShMAX2-CTH

ShHTL7 was labeled with Alexa Fluro 488™ (GREEN dye) in a 1:2 ratio. Then protein–dye mixture was passed through the Sephadex G-25 column to remove the unconjugated dye. The labeled protein (50 nM) was incubated with serial dilutions of ShMAX2-CTH in the absence or presence of 20 µM of GR24 (for 10 min), to determine the *K*_d_ value of the interaction. In another experiment, 100 µM of KK023-N1 was co-incubated with the labeled protein for 2 h, and 20 µM of GR24 was added for 10 min, followed by incubation with a serial dilution of ShMAX2-CTH. About 5 µL of the sample was loaded into premium monolith NT standard capillaries, and the assays were carried out in a NanoTemper Monolith NT.015T instrument. A gradient increase in the temperature was initiated using a laser diode, and the change in the excited fluorescence of the green dye was recorded. To determine the *K*_d_ values of each reaction, the change in the thermophoresis of fluorescent was plotted against the concentrations of un-labeled ShMAX2-CTH, and data were analyzed by NanoTemper software (Hameed et al., 2018).

### GST pull-down of ShHTL7 by CTH of ShMAX2

Purified GST-CTH of ShMAX2 and GST were bound to the Glutathione Sepharose 4B Beads (GE healthcare). Purified 6× His-ShHTL7 was incubated with 100 μM KK023-N1 and Triton X-100 for 3 h, separately. Then apo ShHTL7 was added to the beads bound by GST-CTH of ShMAX2 in the presence and absence of 100 μM GR24. Similarly, ShHTL7 incubated with KK023-N1 and Triton X-100 were added to the beads bound by GST-CTH of ShMAX2 in the presence of 100 μM GR24. Apo ShHTL7 was added to the beads bound by GST only in the presence of 100 μM GR24 as a control. All the samples were washed with lysis buffer (50-mM Tris–HCl pH 8.0, 200 mM NaCl, 2 mM DTT) three times and then eluted with addition of 20 mM reduced glutathione. Thus, the eluted protein was subjected to Western blot using an antibody specific to 6XHis (Abcam, Cambridge, UK).

### DSF assay

About 10 µM of ShHTL7 purified protein was incubated with various concentrations of KK023-N1, Triton X-100, or KK094 (ranging from 0 to 100 µM) for 30 min. Then SYPRO Orange (reporter dye) was added to 1× final concentration. The reactions were carried out in 25 µL 1× PBs buffer. The assay was performed on CFX96 Real-Time System (BIO-RAD, Hercules, CA, USA) thermal cycler on 96-well quantitative polymerase chain reaction (qPCR) white plate, and the plate was sealed with adhesive qPCR plate seal to prevent samples evaporation (Bai et al., 2019). First, samples were heated to 25°C for 1 min followed by linear increase in the temperature ramp from 25°C to 99°C at a rate of 0.5°C/15 s. The change in the protein folding was monitored by the changes in the fluorescence of the reporter dye, and the data were analyzed by the machine software that plots the inflection point of fluorescence against temperature. Data were exported into Excel, and replicates were averaged to generate the graph.

### In silico docking of KK023-N1 to ShHTL7

KK023-N1 was docked to ShHTL7 using online server SwissDock server (Grosdidier et al., 2011). Output results were analyzed to identify the best fitted model with low Gibb’s free energy and biologically relevant structure. The output model was visualized using Pymol, and the final figures were made out of it.

### *Striga* seed germination bioassays

*Striga* seeds were surface sterilized by 20% Sodium hypochlorite (NaClO) for 10 min followed by successive 6 times washing with sterilized MilliQ water. Seeds were kept to dry under the laminar flow cabinet, and ∼50–100 sterilized *Striga* seeds were spread uniformly on a glass fiber filter paper disc (9 mm). For preconditioning, around 12 discs were put in a petri plate on a filter paper moistened with 3 mL sterilized MilliQ water. Plates were then sealed with parafilm and kept in an incubator for 10 d at 30°C (in dark). After preconditioning, plates were removed from the incubator and dried, and discs were pre-treated with different concentrations (0, 5, 10, 20, 40, 80, 160, 320, and 640 μM) of KK023-N1, KK023-N2, Triton X-100, or KK094 for 24 h. Next day, a combination of the inhibitors (same concentrations) along with GR24 (0.5 nM or 1 nM) was applied, and discs were incubated at 30°C for 24 h. For inhibitory effect of KK023-N1 and -N2 on *Striga* seeds obtained from Kenya, seeds were treated with (0, 3, 6, 12, 25, 50,100, 200, 400, and 800 μM). After image scanning, germinated and nongerminated seeds were counted and germination rate (%) was calculated by using SeedQuant software (Braguy et al., 2021).

### Rice germination and growth in response to KK023-N inhibitors

Rice seeds (*cv* Nipponbare) were surface sterilized in 2.5% NaClO and 0.05% of Tween-20 for 10 min, and rinsed 6 times with sterilized MilliQ water. Seeds were imbibed in sterilized water at 30°C for 24 h in the dark. Next day, ∼3 mL of each inhibitor at different concentrations (3, 6, 12, 25, 50, and 100 μM) was added along with 0.5 Murashige and Skoog (MS) medium into 24-well plate, and one imbibed rice seed was placed in each well. The experiment was performed in six replications (one plant/well, *n* = 6) for each concentration. Nontreated rice seeds in 0.5-MS solution were included as a control. Seeds were allowed to germinate at 30°C in the dark for 48 h for monitoring seeds germination, and seeds were moved to a growth chamber with white fluorescent light (130–180 μM m^−2^ s^−1^) and12 h:12 h (Light/Dark (L/D)) period at 28°C. After 1 week, length of rice seedlings was measured and recorded, and pictures of seedlings were taken.

### Rice micro tillering assay

Rice seeds of WT (*cv* Nipponbare) or SL-deficient mutant (*d17)*, were surface sterilized with 2.5% NaClO and 0.05% of Tween-20 for 10 min, and rinsed 6 times with sterilized MilliQ water. About 30 seeds were placed on sterilized Magenta GA-7 plant culture box containing 0.5% MS medium with 0.4% agar and incubated at 30°C in dark. After 2 d, boxes were moved to a growth chamber and allowed to grow for another 5 d under fluorescent white light (130–180 μM m^−2^ s^−1^). Equally grown seedlings were transplanted in 50-mL black tubes filled with Hoagland nutrient solution (1 seedling/tube). Seedlings were treated with 10, 50, and 100 µM of each compound (KK023-N1, Triton X-100, and KK094). The compounds were applied twice a week for 2 weeks to test their effects on the growth of WT plants. SL-deficient seedlings (*d17*) were treated as WT but with the addition of 1 µM GR24. Number of tillers per plant, plant height, and dry biomass were measured at final harvest.

### *Striga* emergence study under greenhouse conditions

Approximately 6,000 *Striga* seeds (20 mg) were weighed, mixed with sand/soil mixture (1:2 ratio), and put into a 3-L perforated plastic pot containing 0.5-L clean sand/soil mixture in the bottom. The pots were kept under greenhouse-controlled conditions at 30°C and kept moist with water for *Striga* preconditioning for 10 d. Formulated KK023-N1 and Triton X-100 were applied (at 100 mL per pot) at 10 and 100 µM concentration. Then three rice seedlings (*cv* IAC165, 10 d old) were planted in each pot after 24 h of inhibitor application. All the treatments were applied continuously twice per week for 4 weeks. Water with same amount of solvent (Cyclohexanone+Atlas-G) was used as a control. Pots were irrigated with 200 mL of Hoagland’s nutrient solution with low phosphate (4 µM) when needed. The rice plants were allowed to grow under greenhouse conditions for 10 weeks and the emerged *Striga* plants were counted from each pot. The height of rice plants was also measured to see impact of *Striga* infestation on host growth.

### Accession numbers

**Table:** 

Gene name	NCBI accession No.
ShHTL7	KR013127.1
ShHTL5	KR013125.1
OsD14	AK070827
ShMAX2	JX565467.1

## Supplemental data  

The following materials are available in the online version of this article.

**Supplemental Figure S1.** NMR analysis of KK023-N1 and KK023-N2.

**Supplemental Figure S2.** ShHTL7/ShHTL5-mediated YLG hydrolysis.

**Supplemental Figure S3.** Effect of KK023-N1 and KK023-N2 on *S. hermonthica* seed germination (Kenya batch).

**Supplemental Figure S4.** Mass spectrometer analysis.

**Supplemental Method S1.** MALDI-TOF and Nano LC–MS. 

**Supplemental Table S1**. Data collection and refinement.

**Supplemental Table S2.** Primer sequences used for cloning ShHTL7/OsD14 and ShHTL5 and ShMAX2-CTH gBlok sequences.

## Supplementary Material

kiab547_Supplementary_Data

## Notes

S.A.B. and T.A. conceived the project. R.A.Z. and U.F.S.H. performed the molecular and in vitro enzymatic assays. U.F.S.H. and S.T.A. performed the crystal structure analysis. R.A.Z., M.J., and J.Y.W. performed pot experiment. M.J. and R.A.Z. performed rice seed germination and growth assays, *Striga* seed germination bioassay, and study the effect of chemicals on rice WT and rice SL deficient mutant (d17) mutant. T.A. and T.O. designed and synthesized KK023-N and KK007 chemical compounds. R.A.Z., M.J., and U.F.S.H. wrote the first draft of the manuscript. All authors contributed to the revising and editing of the manuscript.

The author responsible for distribution of materials integral to the findings presented in this article in accordance with the policy described in the Instructions for Authors (https://academic.oup.com/plphys/pages/general-instructions) is Prof. Salim Al-Babili (salim.babili@kaust.edu.sa).
