# Supplementary material for: Rational design of Striga hermonthica-specific seed germination inhibitors
Source: Plant Physiol. 2021 Nov 27;188(2):1369–84. doi: 10.1093/plphys/kiab547 (PMC8825254; doi:10.1093/plphys/kiab547)
Supplement: kiab547_Supplementary_Data [file kiab547_supplementary_data.zip › Supplemental Files.pptx]

## Slide 1
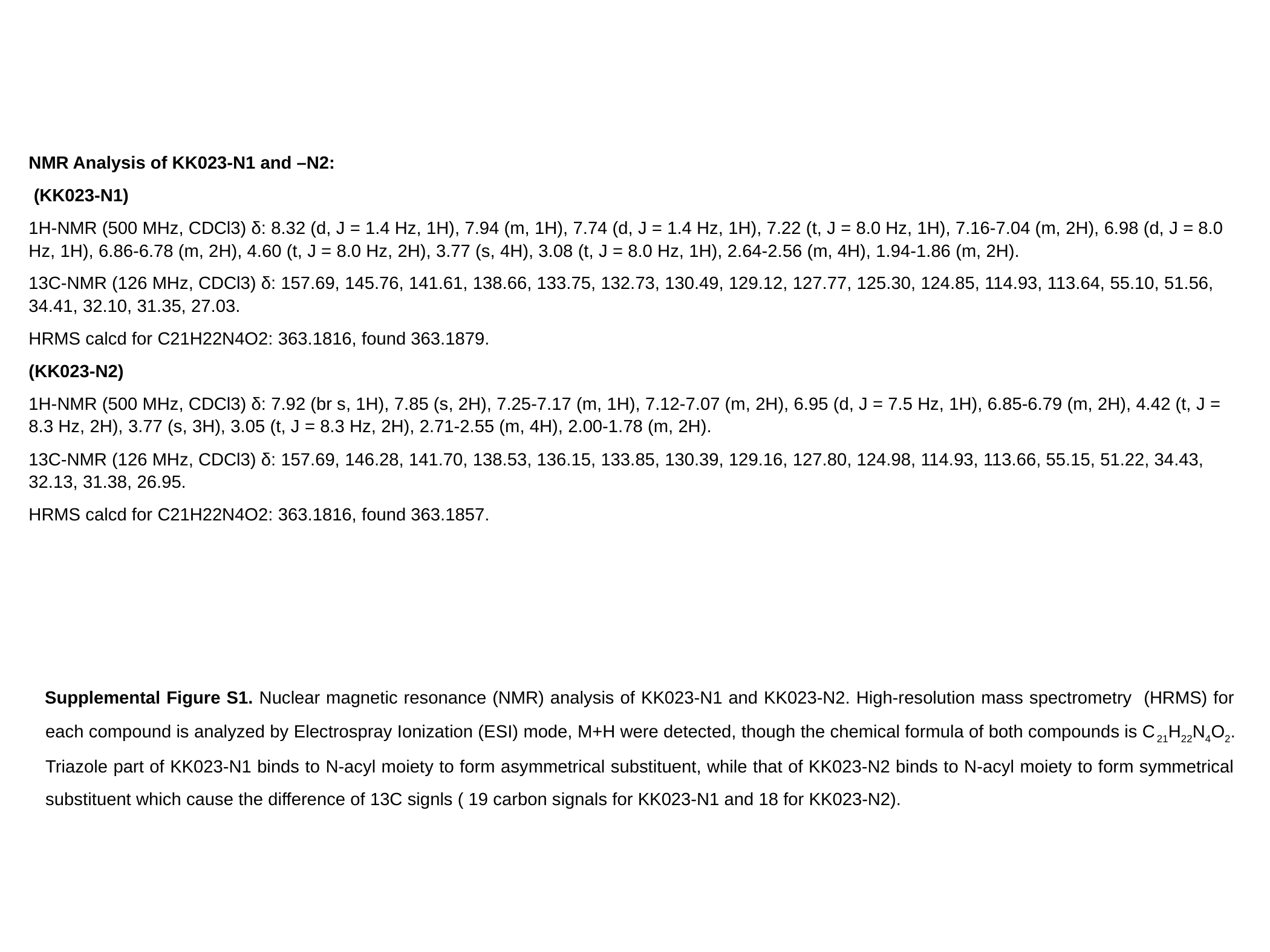

NMR Analysis of KK023-N1 and –N2:
 (KK023-N1)
1H-NMR (500 MHz, CDCl3) δ: 8.32 (d, J = 1.4 Hz, 1H), 7.94 (m, 1H), 7.74 (d, J = 1.4 Hz, 1H), 7.22 (t, J = 8.0 Hz, 1H), 7.16-7.04 (m, 2H), 6.98 (d, J = 8.0 Hz, 1H), 6.86-6.78 (m, 2H), 4.60 (t, J = 8.0 Hz, 2H), 3.77 (s, 4H), 3.08 (t, J = 8.0 Hz, 1H), 2.64-2.56 (m, 4H), 1.94-1.86 (m, 2H).
13C-NMR (126 MHz, CDCl3) δ: 157.69, 145.76, 141.61, 138.66, 133.75, 132.73, 130.49, 129.12, 127.77, 125.30, 124.85, 114.93, 113.64, 55.10, 51.56, 34.41, 32.10, 31.35, 27.03.
HRMS calcd for C21H22N4O2: 363.1816, found 363.1879.
(KK023-N2)
1H-NMR (500 MHz, CDCl3) δ: 7.92 (br s, 1H), 7.85 (s, 2H), 7.25-7.17 (m, 1H), 7.12-7.07 (m, 2H), 6.95 (d, J = 7.5 Hz, 1H), 6.85-6.79 (m, 2H), 4.42 (t, J = 8.3 Hz, 2H), 3.77 (s, 3H), 3.05 (t, J = 8.3 Hz, 2H), 2.71-2.55 (m, 4H), 2.00-1.78 (m, 2H).
13C-NMR (126 MHz, CDCl3) δ: 157.69, 146.28, 141.70, 138.53, 136.15, 133.85, 130.39, 129.16, 127.80, 124.98, 114.93, 113.66, 55.15, 51.22, 34.43, 32.13, 31.38, 26.95.
HRMS calcd for C21H22N4O2: 363.1816, found 363.1857.
Supplemental Figure S1. Nuclear magnetic resonance (NMR) analysis of KK023-N1 and KK023-N2. High-resolution mass spectrometry (HRMS) for each compound is analyzed by Electrospray Ionization (ESI) mode, M+H were detected, though the chemical formula of both compounds is C21H22N4O2. Triazole part of KK023-N1 binds to N-acyl moiety to form asymmetrical substituent, while that of KK023-N2 binds to N-acyl moiety to form symmetrical substituent which cause the difference of 13C signls ( 19 carbon signals for KK023-N1 and 18 for KK023-N2).

## Slide 2
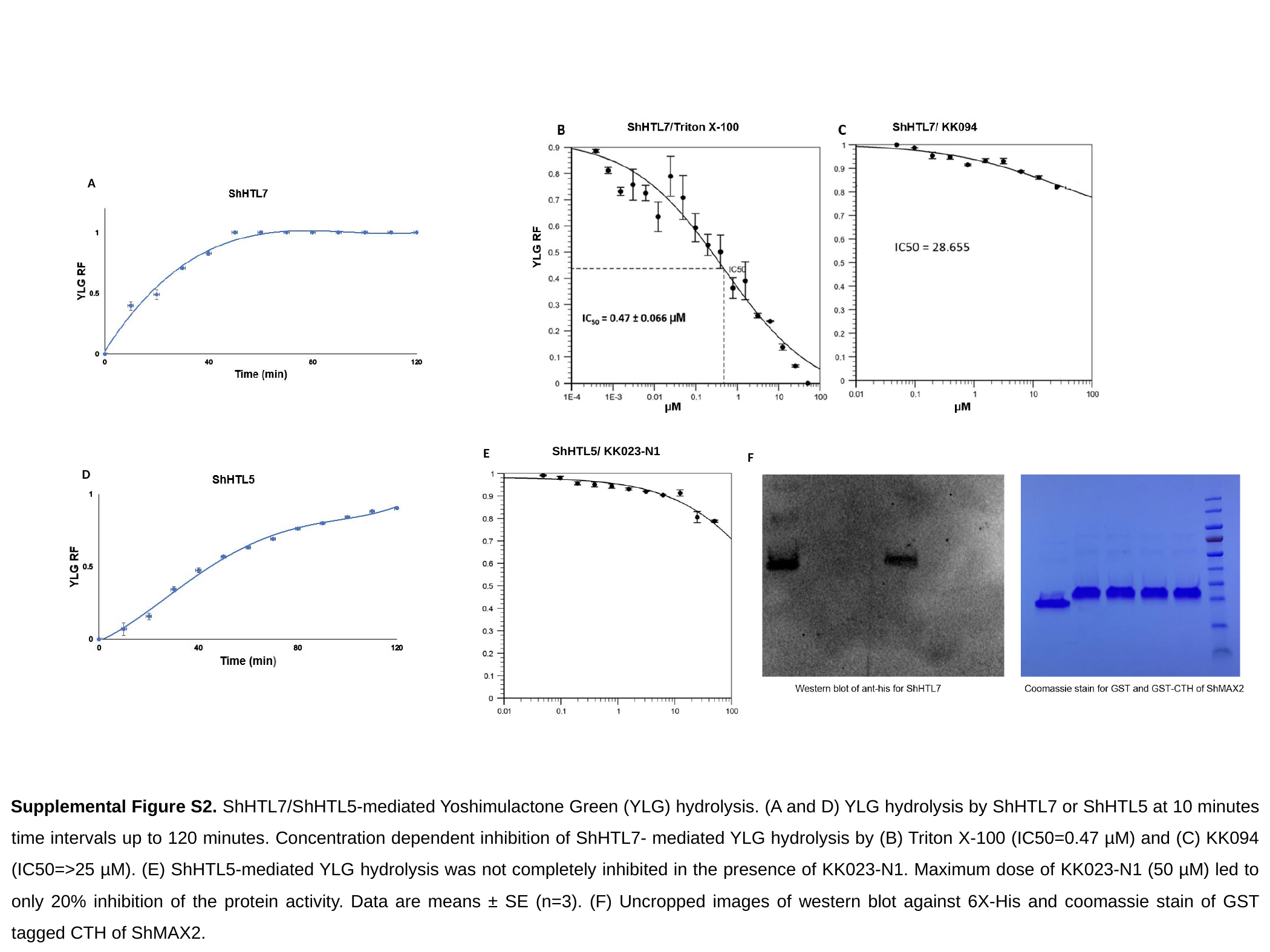

A
ShHTL5/ KK023-N1
E
F
D
µM
Supplemental Figure S2. ShHTL7/ShHTL5-mediated Yoshimulactone Green (YLG) hydrolysis. (A and D) YLG hydrolysis by ShHTL7 or ShHTL5 at 10 minutes time intervals up to 120 minutes. Concentration dependent inhibition of ShHTL7- mediated YLG hydrolysis by (B) Triton X-100 (IC50=0.47 µM) and (C) KK094 (IC50=>25 µM). (E) ShHTL5-mediated YLG hydrolysis was not completely inhibited in the presence of KK023-N1. Maximum dose of KK023-N1 (50 µM) led to only 20% inhibition of the protein activity. Data are means ± SE (n=3). (F) Uncropped images of western blot against 6X-His and coomassie stain of GST tagged CTH of ShMAX2.

## Slide 3
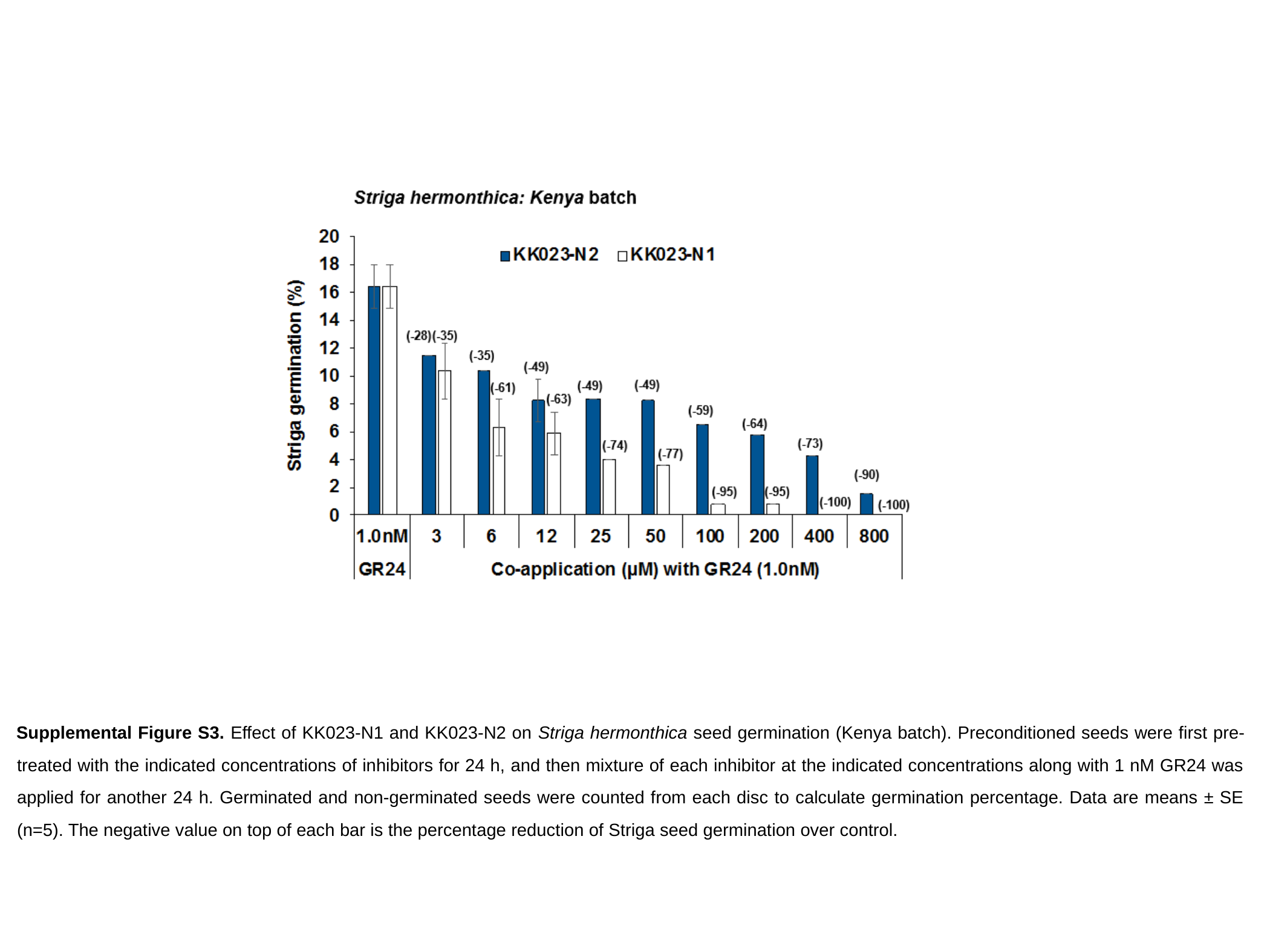

Supplemental Figure S3. Effect of KK023-N1 and KK023-N2 on Striga hermonthica seed germination (Kenya batch). Preconditioned seeds were first pre-treated with the indicated concentrations of inhibitors for 24 h, and then mixture of each inhibitor at the indicated concentrations along with 1 nM GR24 was applied for another 24 h. Germinated and non-germinated seeds were counted from each disc to calculate germination percentage. Data are means ± SE (n=5). The negative value on top of each bar is the percentage reduction of Striga seed germination over control.

## Slide 4
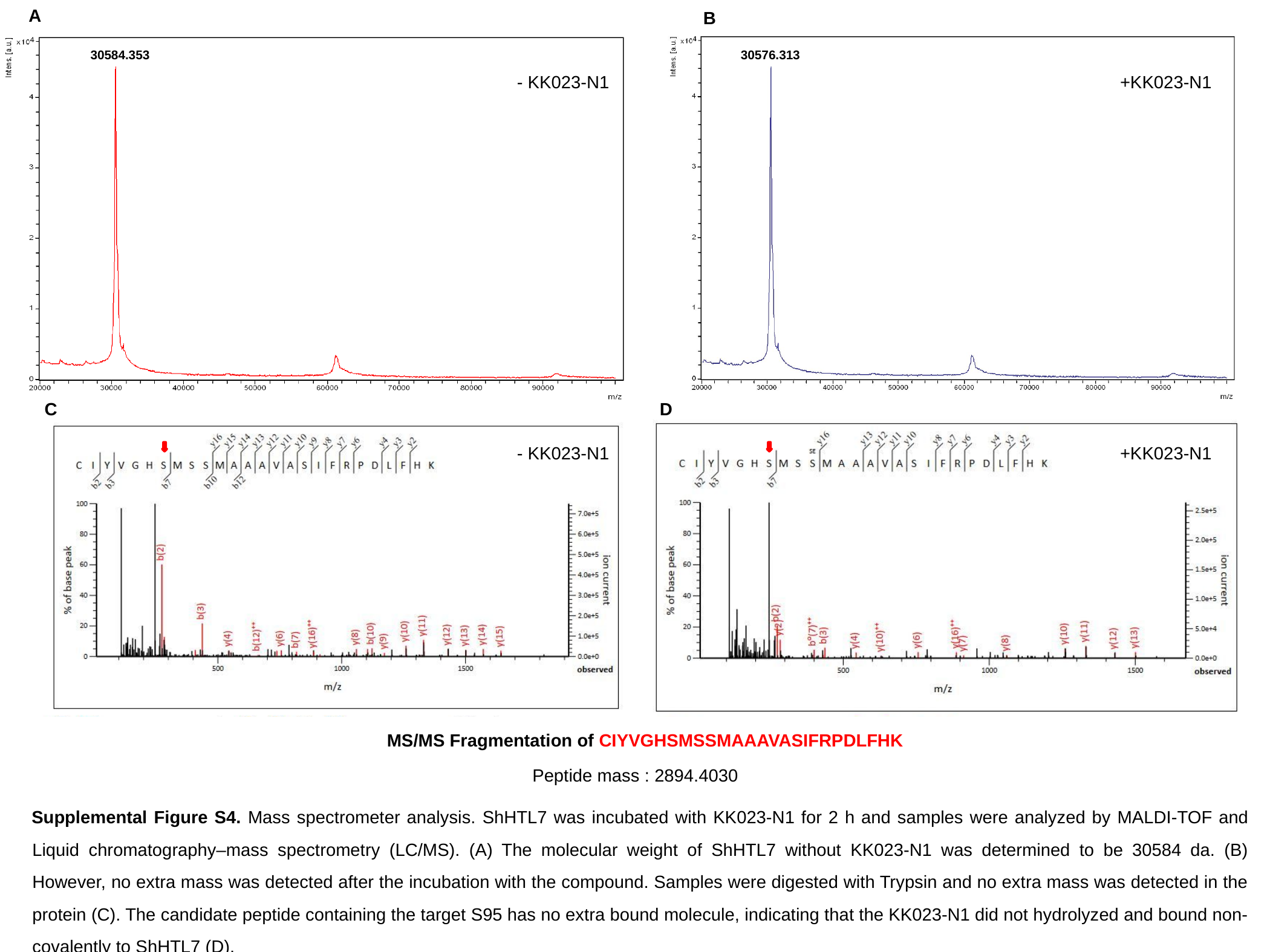

A
B
30576.313
30584.353
- KK023-N1
+KK023-N1
B
D
C
- KK023-N1
+KK023-N1
 MS/MS Fragmentation of CIYVGHSMSSMAAAVASIFRPDLFHK
Peptide mass : 2894.4030
Supplemental Figure S4. Mass spectrometer analysis. ShHTL7 was incubated with KK023-N1 for 2 h and samples were analyzed by MALDI-TOF and Liquid chromatography–mass spectrometry (LC/MS). (A) The molecular weight of ShHTL7 without KK023-N1 was determined to be 30584 da. (B) However, no extra mass was detected after the incubation with the compound. Samples were digested with Trypsin and no extra mass was detected in the protein (C). The candidate peptide containing the target S95 has no extra bound molecule, indicating that the KK023-N1 did not hydrolyzed and bound non-covalently to ShHTL7 (D).

## Slide 5
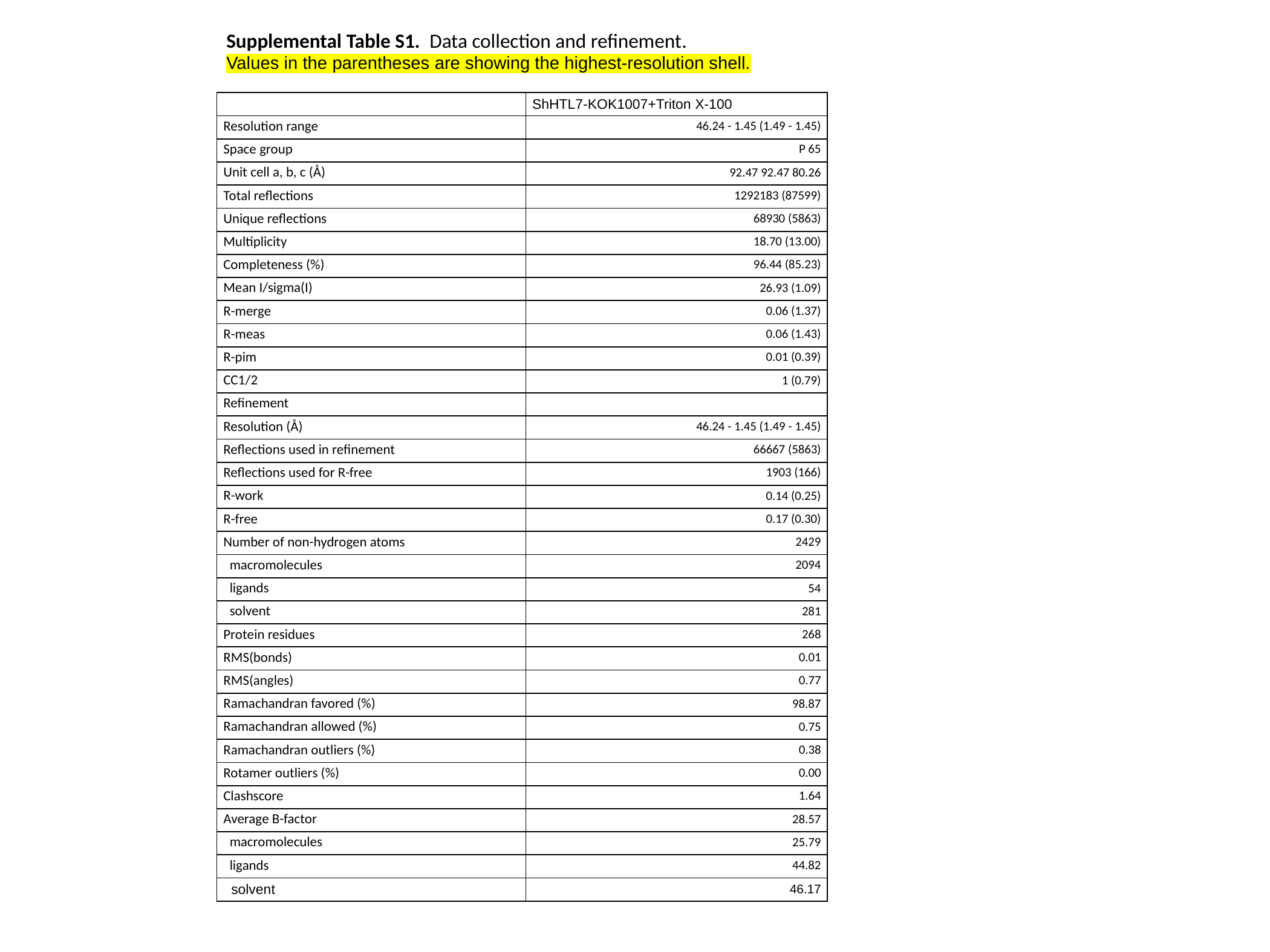

Supplemental Table S1. Data collection and refinement.
Values in the parentheses are showing the highest-resolution shell.
| | ShHTL7-KOK1007+Triton X-100 |
| --- | --- |
| Resolution range | 46.24 - 1.45 (1.49 - 1.45) |
| Space group | P 65 |
| Unit cell a, b, c (Å) | 92.47 92.47 80.26 |
| Total reflections | 1292183 (87599) |
| Unique reflections | 68930 (5863) |
| Multiplicity | 18.70 (13.00) |
| Completeness (%) | 96.44 (85.23) |
| Mean I/sigma(I) | 26.93 (1.09) |
| R-merge | 0.06 (1.37) |
| R-meas | 0.06 (1.43) |
| R-pim | 0.01 (0.39) |
| CC1/2 | 1 (0.79) |
| Refinement | |
| Resolution (Å) | 46.24 - 1.45 (1.49 - 1.45) |
| Reflections used in refinement | 66667 (5863) |
| Reflections used for R-free | 1903 (166) |
| R-work | 0.14 (0.25) |
| R-free | 0.17 (0.30) |
| Number of non-hydrogen atoms | 2429 |
| macromolecules | 2094 |
| ligands | 54 |
| solvent | 281 |
| Protein residues | 268 |
| RMS(bonds) | 0.01 |
| RMS(angles) | 0.77 |
| Ramachandran favored (%) | 98.87 |
| Ramachandran allowed (%) | 0.75 |
| Ramachandran outliers (%) | 0.38 |
| Rotamer outliers (%) | 0.00 |
| Clashscore | 1.64 |
| Average B-factor | 28.57 |
| macromolecules | 25.79 |
| ligands | 44.82 |
| solvent | 46.17 |

## Slide 6
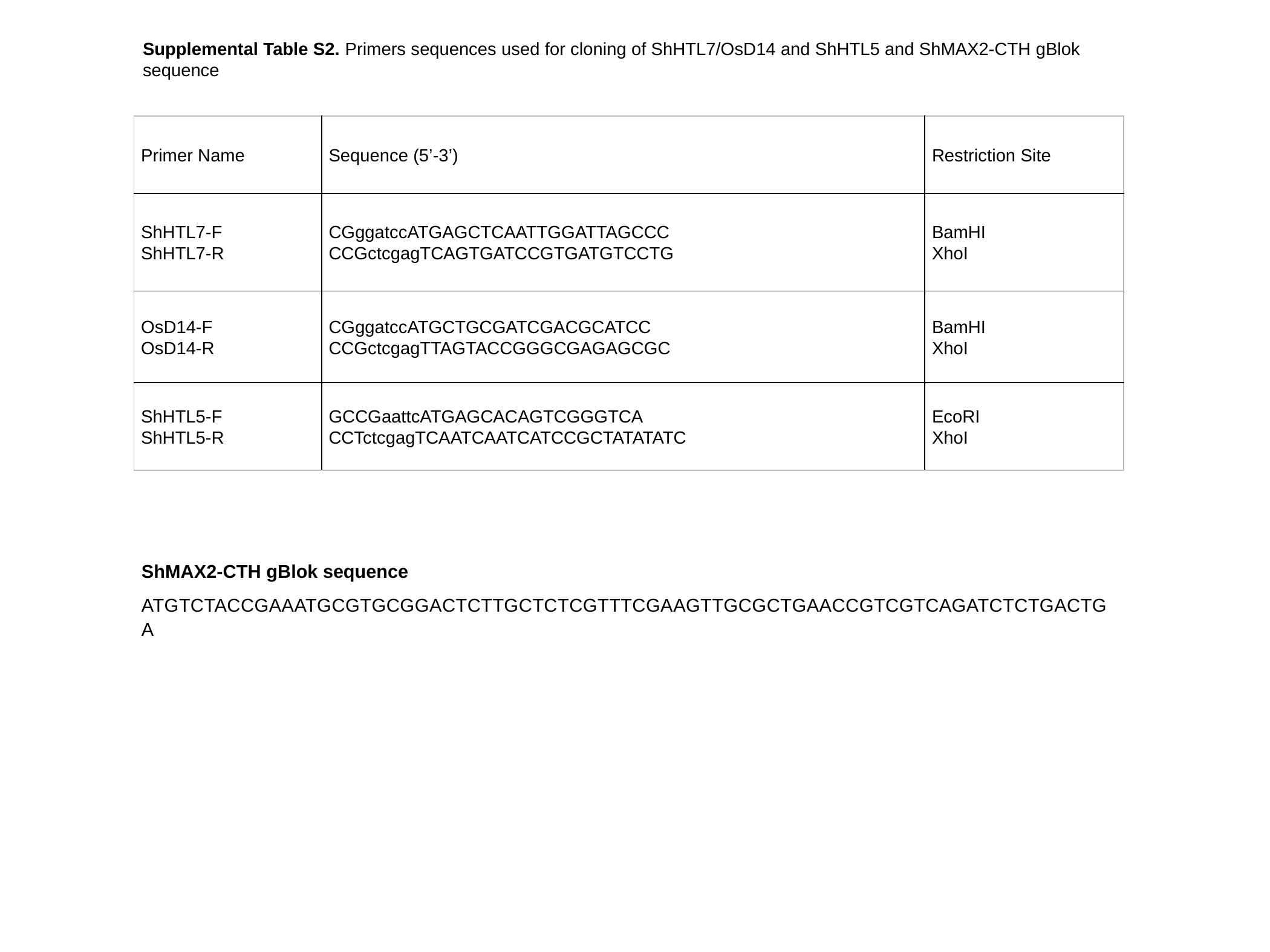

Supplemental Table S2. Primers sequences used for cloning of ShHTL7/OsD14 and ShHTL5 and ShMAX2-CTH gBlok sequence
| Primer Name | Sequence (5’-3’) | Restriction Site |
| --- | --- | --- |
| ShHTL7-F ShHTL7-R | CGggatccATGAGCTCAATTGGATTAGCCC CCGctcgagTCAGTGATCCGTGATGTCCTG | BamHI XhoI |
| OsD14-F OsD14-R | CGggatccATGCTGCGATCGACGCATCC CCGctcgagTTAGTACCGGGCGAGAGCGC | BamHI XhoI |
| ShHTL5-F ShHTL5-R | GCCGaattcATGAGCACAGTCGGGTCA CCTctcgagTCAATCAATCATCCGCTATATATC | EcoRI XhoI |
ShMAX2-CTH gBlok sequence
ATGTCTACCGAAATGCGTGCGGACTCTTGCTCTCGTTTCGAAGTTGCGCTGAACCGTCGTCAGATCTCTGACTGA

## Slide 7
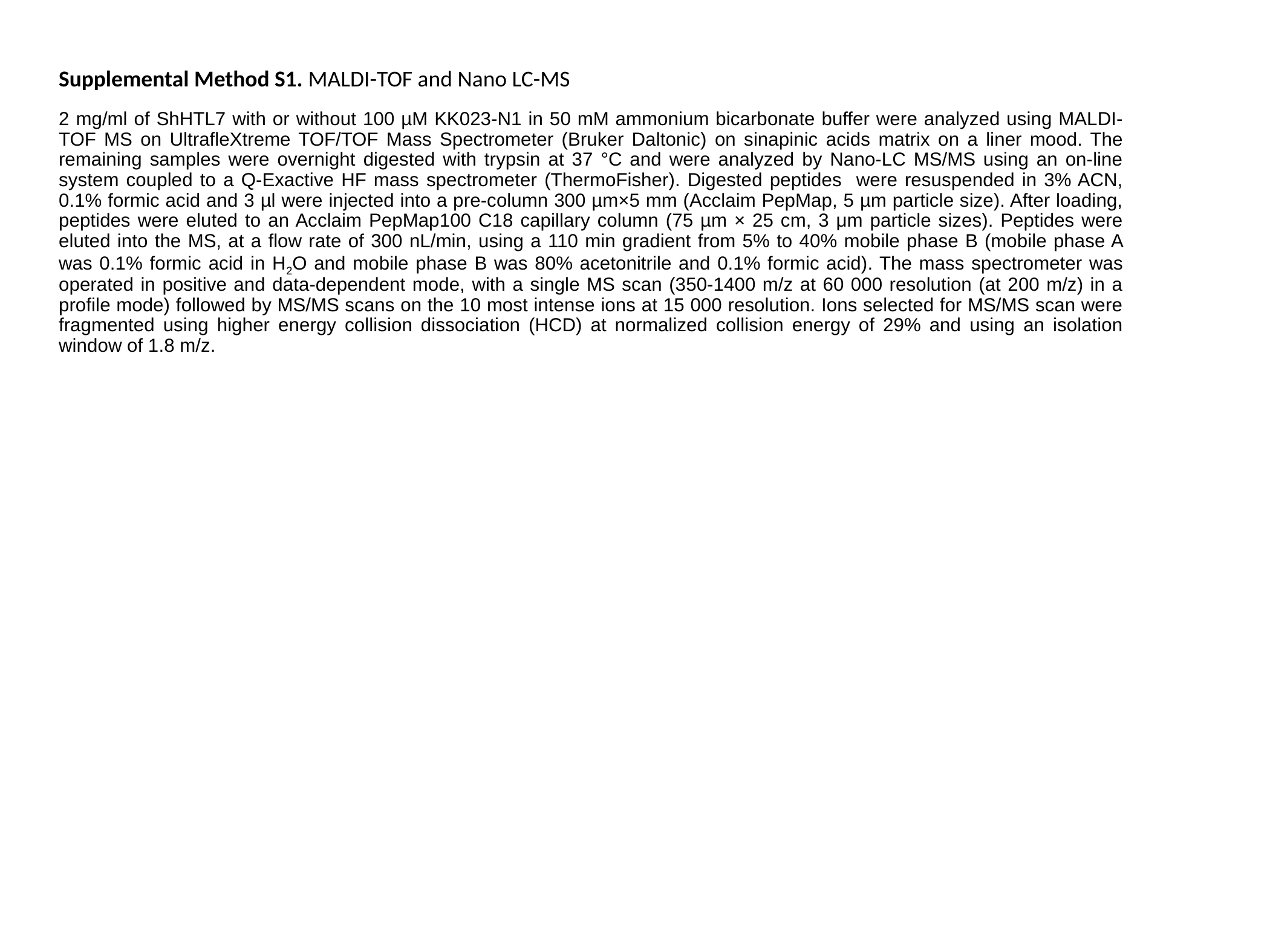

Supplemental Method S1. MALDI-TOF and Nano LC-MS
2 mg/ml of ShHTL7 with or without 100 µM KK023-N1 in 50 mM ammonium bicarbonate buffer were analyzed using MALDI-TOF MS on UltrafleXtreme TOF/TOF Mass Spectrometer (Bruker Daltonic) on sinapinic acids matrix on a liner mood. The remaining samples were overnight digested with trypsin at 37 °C and were analyzed by Nano-LC MS/MS using an on-line system coupled to a Q-Exactive HF mass spectrometer (ThermoFisher). Digested peptides were resuspended in 3% ACN, 0.1% formic acid and 3 µl were injected into a pre-column 300 µm×5 mm (Acclaim PepMap, 5 µm particle size). After loading, peptides were eluted to an Acclaim PepMap100 C18 capillary column (75 µm × 25 cm, 3 μm particle sizes). Peptides were eluted into the MS, at a flow rate of 300 nL/min, using a 110 min gradient from 5% to 40% mobile phase B (mobile phase A was 0.1% formic acid in H2O and mobile phase B was 80% acetonitrile and 0.1% formic acid). The mass spectrometer was operated in positive and data-dependent mode, with a single MS scan (350-1400 m/z at 60 000 resolution (at 200 m/z) in a profile mode) followed by MS/MS scans on the 10 most intense ions at 15 000 resolution. Ions selected for MS/MS scan were fragmented using higher energy collision dissociation (HCD) at normalized collision energy of 29% and using an isolation window of 1.8 m/z.
